# New Approaches to Dendritic Cell-Based Therapeutic Vaccines Against HIV-1 Infection

**DOI:** 10.3389/fimmu.2021.719664

**Published:** 2022-01-04

**Authors:** Marisierra Espinar-Buitrago, Ma Angeles Muñoz-Fernández

**Affiliations:** ^1^ Section Head Immunology, Laboratorio InmunoBiología Molecular, Hospital General Universitario Gregorio Marañón (HGUGM), Madrid, Spain; ^2^ Instituto de Investigación Sanitaria Gregorio Marañón (IiSGM), Madrid, Spain; ^3^ Spanish Human Immunodeficiency Virus- Hospital Gregorio Marañón (HIV-HGM) BioBank, Madrid, Spain; ^4^ Networking Research Center on Bioengineering, Biomaterials and Nanomedicine (CIBER-BBN), Madrid, Spain

**Keywords:** therapeutic vaccines, nanotechnology, T-cell response, dendritic cell, HIV-1

## Abstract

Due to the success of combined antiretroviral therapy (cART) in recent years, the pathological outcome of Human Immunodeficiency Virus type 1 (HIV-1) infection has improved substantially, achieving undetectable viral loads in most cases. Nevertheless, the presence of a viral reservoir formed by latently infected cells results in patients having to maintain treatment for life. In the absence of effective eradication strategies against HIV-1, research efforts are focused on obtaining a cure. One of these approaches is the creation of therapeutic vaccines. In this sense, the most promising one up to now is based on the establishing of the immunological synapse between dendritic cells (DCs) and T lymphocytes (TL). DCs are one of the first cells of the immune system to encounter HIV-1 by acting as antigen presenting cells, bringing about the interaction between innate and adaptive immune responses mediated by TL. Furthermore, TL are the end effector, and their response capacity is essential in the adaptive elimination of cells infected by pathogens. In this review, we summarize the knowledge of the interaction between DCs with TL, as well as the characterization of the specific T-cell response against HIV-1 infection. The use of nanotechnology in the design and improvement of vaccines based on DCs has been researched and presented here with a special emphasis.

## Introduction

Human Immunodeficiency Virus type 1 (HIV-1) continues to be a health problem worldwide, particularly in developing countries. Although the proportion of the population infected with the virus has stabilized since 2000, the total number of infected subjects continues to increase. According to UNAIDS data from 2019, 38 million people are living with HIV-1, increasing the number of new infections to 1.7 million people in that year.

The discovery of antiretroviral therapies, especially the cATR regimen, has helped the progression of the infection from a fatal to a chronic disease. Access and adherence to treatment are determining factors for the good medical prognosis of patients with this infection (1). However, both factors can be easily lost due to economic problems, social stigmas, and psychological side effects, especially in less developed countries. Despite deaths related to AIDS having drastically decreased in developed countries, many associated co-morbidities, commonly known as non-AIDS-events, decrease the quality of life of the infected and are the first cause of death in HIV populations, according to the CROI 2020 data. Therefore, a new long-term therapeutic/preventive approach against HIV-1 infection must be found (2).

In the absence of a complete cure for HIV-1 infection, a functional cure appears to be the most promising option (3). The definition of a functional cure states that, although the virus is still present in the host organism and remains latent in the genome of many cells as a reservoir, the immune system keeps the infection under control in the absence of cART (4).

The viral latency, the high mutability, and the variability of this virus make the search for a therapeutic cure extremely difficult. In this sense, the scientific community has spent many years working intensively to achieve this aim through different strategies, including the creation of vaccines against HIV-1. Therapeutic vaccines could contribute to HIV-1 therapy as their application immediately after the infection could limit the size of the virus reservoir and prevent future viral spread (5).

A significant number of vaccine candidates have been tested and failed. The main mechanism of all these vaccines is a delivery of HIV-1 antigenic peptides to antigen presenting cells (APC) to obtain a therapeutic response (6, 7).

## Spread of Virus in the Immune System

### HIV-1 Entry and Processing Inside Dendritic Cells

**Figure 1:**
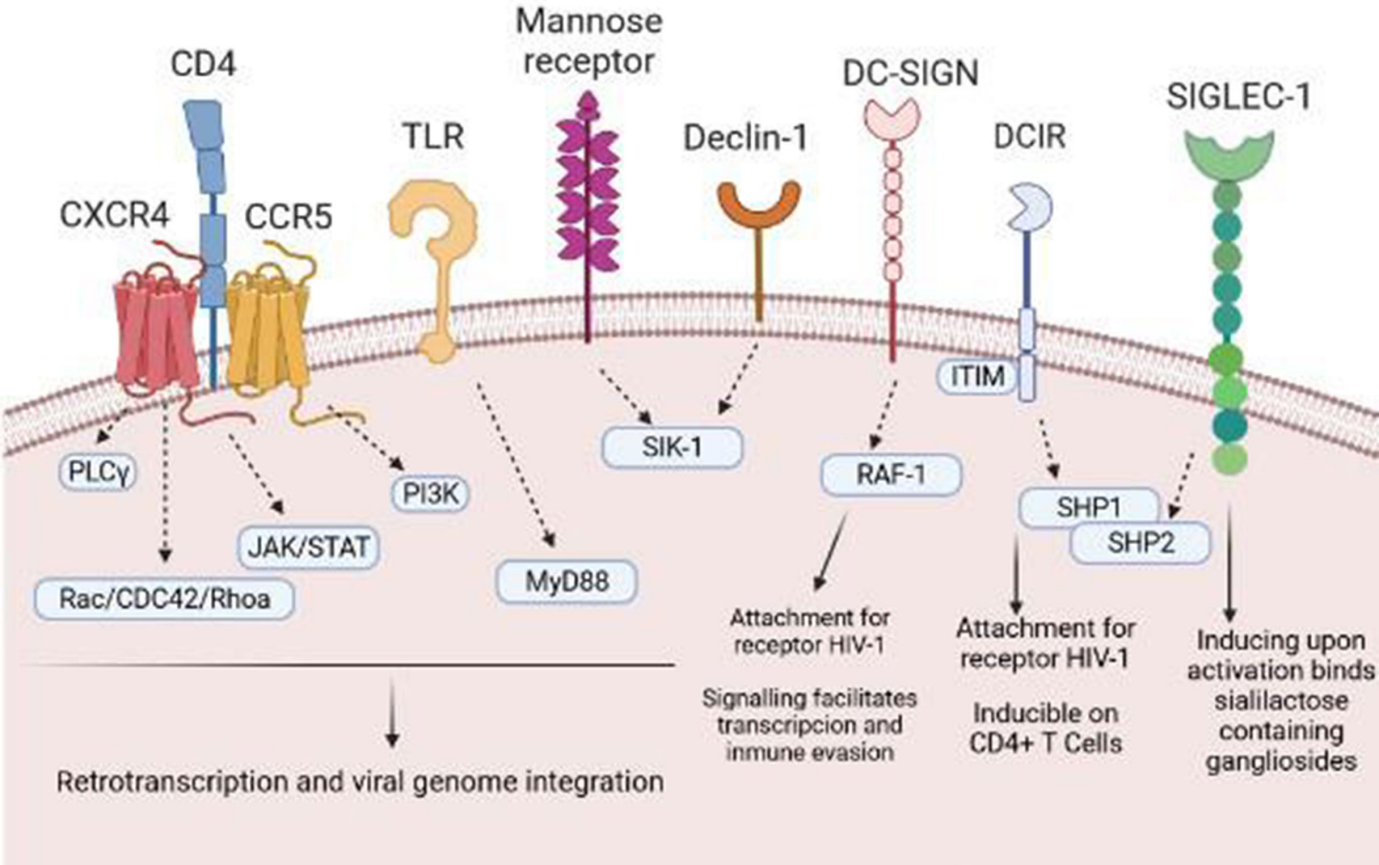
Receptors and pathways involved in the entry of HIV-1 into DCs. HIV-1 binds to several different DC surface receptors, determining the fate of the virus. Generally, HIV-1 is introduced to DCs through endocytosis after binding to DC-SIGN or other receptors, such as Declin-1 or Siglec-1. Binding to these receptors generates a series of intracellular signals that allow transinfection or immunological recognition and the consequent activation of T cells.

HIV-1 is capable of reaching a wide variety of immune cells. In this sense, the contact of the virus with the target TLs is an important mechanism for establishing a persistent infection. However, the first step is the arrival of the virus to APCs, in this case DCs, which will transmit HIV-1 to TLs (8). HIV-1 can use different receptors/binding pathways to infect DCs, including the CCR5 receptor, the CXC4 chemokine receptor (CXCR4), or C lectin type receptors, especially DC-SIGN (dendritic cell-specific ICAM-3 grabbing nonintegrin) that bind to the HIV-1 envelope, specifically, to gp120 (9). Then, the virus can transmit to the target cell or degrade in endosomes by binding to langergin before transmission occurs (10). In addition to this receptor dependent on the binding of gp120, Singlec-1 or CD169 is another receptor independent of binding to this glycoprotein. Singlec-1 is expressed in DCs; specifically it is found within the uropodia of migratory DCs, facilitating the capture and retention of HIV-1 by binding to ganglioside GM3 (11) **(** **Figure 1** **)**. Furthermore, Singlec-1 has the quality of being within the compartments of DCs, and TLs can access to them in order to improve the interaction between DCs with TLs. In addition to this glycoprotein-dependent viral capture by DCs, HIV-1 can also be taken up by a lipid-dependent mechanism (12, 13). Moreover, HIV-1 has the ability to incorporate other virus envelope glycoproteins during assembly, a phenomenon known as pseudotyping. This allows HIV-1 to expand its cellular tropism and enables it to infect not only T cells (14).

The process of interaction between viral particles and DCs generates a series of responses that lead to the maturation and regulation of chemokine receptors, which will help in the migration process to secondary lymphoid organs to initiate the adaptive immune response. DCs have been shown to undergo activation and maturation through different virus-induced mechanisms (15). These pathways of activation and maturation include those mediated by toll like receptor (TLR), the stimulator of the interferon gene (STING) (16), the cyclic pathway of guanosine monophosphate-adenosine monophosphate synthase (cGAS) (17), and the mitochondrial pathway (MAVs) (18). Despite HIV-1 infection, DCs can maintain their ability to migrate to the lymph nodes under the influence of environmental chemotactic factors such as sphingosine 1-phosphate (S1P) and CCL19/21, so DCs expression remains unaltered (19, 20).

Once the DCs reach the lymphoid organs, contact with the T cells begins. Although the molecular mechanisms are still not entirely clear, it is known that DCs capture the antigen by endocytosis, degrade it to peptides after endosome-lysosome fusion, and present it to T cells in the context of MHC (21). The union established between the antigen and MHC, with the collaboration of costimulatory signals on the surface of the DCs, forms an environment known as “immunological synapse”, capable of inducing the specific T response and, therefore, the adaptive immune response (22).

For optimal antigen presentation to T cells, DCs must execute good intracellular processing. However, cells infected with HIV-1 present the antigen through MHC-I, through cross-presentation (23). It is well known that HIV-1 exploits multiple mechanisms to evade immune recognition, including a high mutation rate, glycosylation of the envelope protein gp120, or in this case the virus has the ability to manipulate host antigen presentation and processing mechanisms (24).

Different HIV-1 proteins are involved in modifying these antigen presentation pathways so virus seeks the endosomal pathway, avoiding the lysosomal pathway, becoming a proliferating way for the virus to spread to other places such as the lymph nodes (24, 25). Endosomal processing may provide another opportunity to escape immunological recognition by promoting the destruction of key antigens by endosomal proteases. During cross-presentation, antigens are exposed to endosomal proteases. In this sense, the HIV-1 Nef protein interrupts the presentation of antigens on the cell surface by interfering with the normal trafficking pathway of MHC-I, through the AP-1-mediated signaling. This mechanism reduces the recognition of cytotoxic T lymphocytes and the lysis of infected cells, using an immune escape route (26, 27).

### Surface DCs-T Cells Interactions in the Presence of HIV-1

During the immune synapse, T cells are able to probe the surface of the protrusions generated by DCs, inside which there is a large viral load. Due to exploratory contacts, the virus particles produced from these DCs can be transferred to T cells with high efficiency. In this context, a relatively small amount of DCs containing viral particles can generate an increase in T-cell infection, making it exponential (28, 29).The number of interactions established between DCs and TLs, as well as how long they remain in contact, cause T cells differentiation and acquisition of the ability to carry out effector and memory responses. Memory T cells can establish themselves as a reservoir during the chronic phase of viral infection, so that these target cells can cause virus reactivation favoring dissemination (30). A recent study in people with chronic HIV-1 infection has shown that CD8+ T cells not only responded to mutated HIV-1 epitopes which cause death of CD4+ T cells, but also led to increased maturation of cells producing higher transinfection of CD4+ T cells (31). The persistence of mutated viral strains gives HIV-1 an advantage over the immune system.

**Figure 2:**
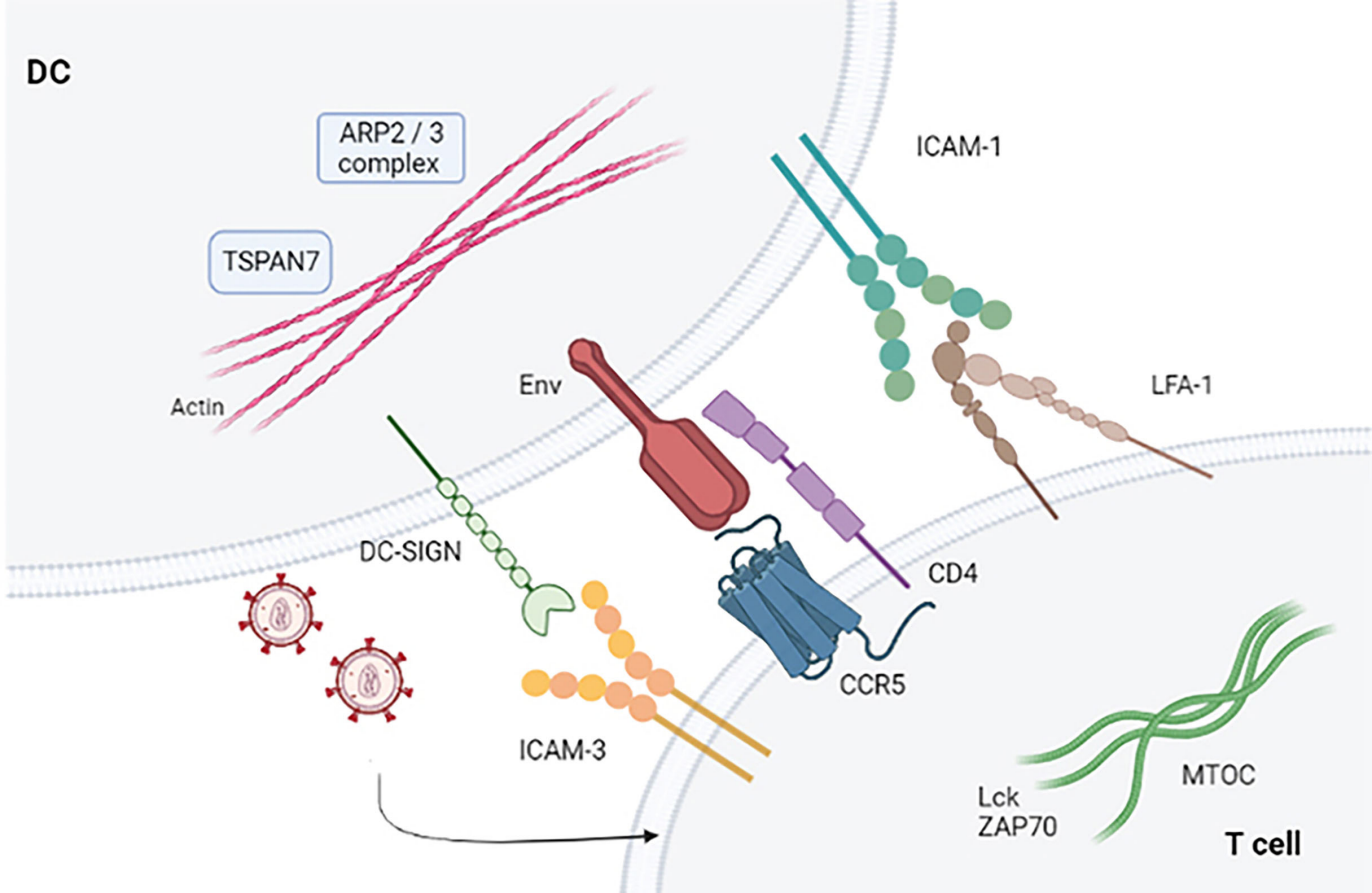
Virological synapse in the presence of HIV-1. Virological synapse in the presence of HIV. The interaction between the different receptors present in both DCs and T cells allows entry into the target cells (CD4+ T and CD8+ T lymphocytes), thus allowing the dissemination of HIV-1 that escapes the immune response mechanism produced by these cells.

When T cells establish contact with viral particles embedded within membrane invaginations, a signaling cascade occurs that ends when T cells stop binding DCs. Knowledge of cell dynamics that orchestrate viral shedding from DCs to T cells is still lacking. HIV-1 binds to accessible surface adhesion and signaling receptors, forming a crucial point of contact with T cells. This leads to activation mediated by gp120 and lymphocyte function-associated antigen 1 (LFA-1) that initiates signal transduction by inducing T-cell entrapment and activation (32, 33). The interaction between DCs and T cells allows trapped HIV-1 particles to reach target T cells in a manner similar to that established in DCs, wherein DCs can capture ganglioside-rich exosomes (34). It has been shown that increasing numbers of viruses can bind directly to integrins (35). Particular importance is the adhesive interaction between LFA-1 and ICAM-1 that facilitate virologic synapse and cell-to-cell transmission (36). Blocking the binding between LFA1 and ICAM-1 prevents prolonged contact between DCs and T cells. Therefore, the established binding between gp120 and T cells is necessary for activation of LFA-1 (37). LFA-1 is activated when the binding of the gp120 protein to α4β7 occurs in primary T cells. The virological synapse is closely related to the activation of LFA-1, as well as to the signals that are established in the interaction of gp120 through α4β7 (38, 39). Therefore, the binding between the virus glycoprotein and T cells represents a limiting step in facilitating prolonged contacts of DCs and T cells (40) **(** **Figure 2** **)**.

The ability to capture and transmit viral particles possessed by DCs and their presentation to T cells at the site of cell-to-cell contact, known as transinfection, promotes systemic release after viral exposure (41, 42).

In addition to the interaction of the virus with the integrins, there is another family of proteins with a very important role in the transinfection process. Tetraspanins family is responsible for regulating intracellular traffic and modulating the function of other helper molecules, controlling their expression in the plasma membrane or classifying themselves into intracellular vesicles. Tetraspanins interact with other members of the superfamily, other transmembrane receptors, lipids, signaling molecules, and cytoskeletal components (43, 44). These proteins attend to the organization of tetraspanin-enriched microdomains (TEM) containing CD9, CD63, CD81, and CD82 in macrophages, DCs, and T cells (45). TEMs regulate the recognition and presentation of Ag and the activation and proliferation of T cells, as well as the extravasation of leukocytes associating with MHC-I and MHC-II on the surface of DCs (46). HIV-1 uses the mechanisms provided by the host cells to its advantage. Individually, CD63 has a dual function (26, 27, 47). On the one hand, it is essential for the replication of HIV-1 together with the CXCR4 and CCR5 receptor on T cells participating in virus entry or reverse transcription, and on the other hand, the expression of CD63 supports mediated fusion by Env during transduction, so it regulates the replication step. However, its activity and function have not yet been fully understood (48). In addition, CD9, CD63, and CD81 are involved in the immunological synapse at virion-enriched budding sites (49). CD81 is important in cross-presentation of antigen by DCs to cytotoxic T cells. In addition, it is capable of increasing the availability of dNTPs and therefore favoring HIV-1 infection thanks to the blocking SAMHD1, a restriction factor that allows DCs to escape productive HIV-1 infection (50). CD9 and CD81 are found in extracellular vesicles as exosomes. Blocking TEMs has previously been shown to result in a significant decrease in exosomal uptake efficiency in DCs (51). Both CD9 and CD81 bind to the cytoskeleton through proteins of the ezrin-radixin-moeisin family (ERM) which bind to actin, a key element in the interaction between DCs and TLs and in propagation of HIV-1 (52, 53). There is a membrane protein from tetraspanin family that, associated with the ARP2/3 complex, is capable of promoting nucleation and stabilizing actin. This protein is TSPAN7. TSPAN7 prevents the viral particles from being internalized, remaining close to the membrane of the DCs, sites rich in actin, favoring the dissemination to T cells (54).

### Intracellular Signaling Pathways

The different interactions that are established between protein families during the virological synapse generate an intracellular signaling cascade (55). This activation induces the reorganization of MOTC and T cells polarization in cell-cell contact sites (56). HIV-1 infection of T cells occurs during the acute phase. One question is if virologic synapse favors the transmission of HIV-1 particles to T cells. Recent studies have shown that clustering of the viral Env protein with TLs initiated T cell receptor signaling, enhancing the transmission of HIV-1 between cells (57). Furthermore, signal amplification occurred in these T cells, suggesting that *de novo* expression of Env in infected T cells was able to initiate further activation signals during cell-cell contacts (58). The Env protein of HIV-1 induces a calcium flux which initiates an intracellular signaling cascade that produces MEK/ERK activation which induces phosphorylation of Lck and partial activation of ZAP-70, a tyrosine kinase protein from SyK family which plays a critical factor in T cell signaling (59).

These tyrosine kinases accomplish an essential function for T cells since they intervene in their maturation and differentiation. The Tec proteins trigger a signaling cascade in which LTK is phosphorylated and as a consequence the mobilization of Ca2 + occurs. Ca2+ involves the mobilization of actin responsible for cytoskeletal reorganization (60). This phosphorylation of LTK occurs after contact of ICAM-1 integrin with the gp120 protein in the presence of HIV-1 (61, 62). However, low levels of LTK activation occur, so the virus prevents actin from performing and reduces intracellular p24 levels. This suggests that the signaling pathway would be related to the transcription of the virus (63). Also, the interaction between the activation of the glycoprotein and the CD4+ T cells activates the PI3K pathway responsible for regulating the migration of T cells and promoting the entry of viral particles after HIV-1 infection (64). Furthermore, ERK phosphorylation is needed for reverse transcription, regulating virus infectivity (65, 66). Signaling is dependent on chemokine receptors, but these signals converge to regulate the behavior and function of T cell migration.

Another very important point at the intracellular signaling level is related to the ability of HIV-1 to spread to other cells. In this context, a little-studied family of proteins, the ESCRT, has great relevance (67). These will form cytosolic complexes establishing themselves as a machinery of remodeling both the cytoplasm and the cell membrane, allowing the viral budding process (68, 69). HIV-1 is able to recruit this machinery upon activation of ALIX and Tsg101 to mediate the budding process (70, 71). Furthermore, it seems that the ESCRT machinery is closely involved with the antigen presenting function of DCs by MHC-II (72). On the one hand, in non-activated DCs, ESCRT is capable of transporting antigens from MHC-II to phagolysosomes. On the other, when DCs have undergone activation, it supports the antigenic presentation process (73, 74).

There is a wide variety of signaling pathways connected and formed by mediators that intervene in multiples cellular processes, and are involved in viral dynamics. The study of these secondary effectors expand diverse treatment design strategies (75). One of the new discoveries that has been recently studied is lymphocyte activation pathway that does not require the recognition and presentation of antigens by the different APCs. This opens the possibility of a new pathway that could act in parallel or not from a canonical one (76).

Thereby, the antigenic presentation process in DCs and the consequent activation of T cells are a true platform to design new therapeutic vaccines. Knowledge of both the signaling pathways and all the participating intermediate molecules is essential to achieve the objective of an effective therapeutic vaccine.

## Vaccines Based on DCs

**Figure 3:**
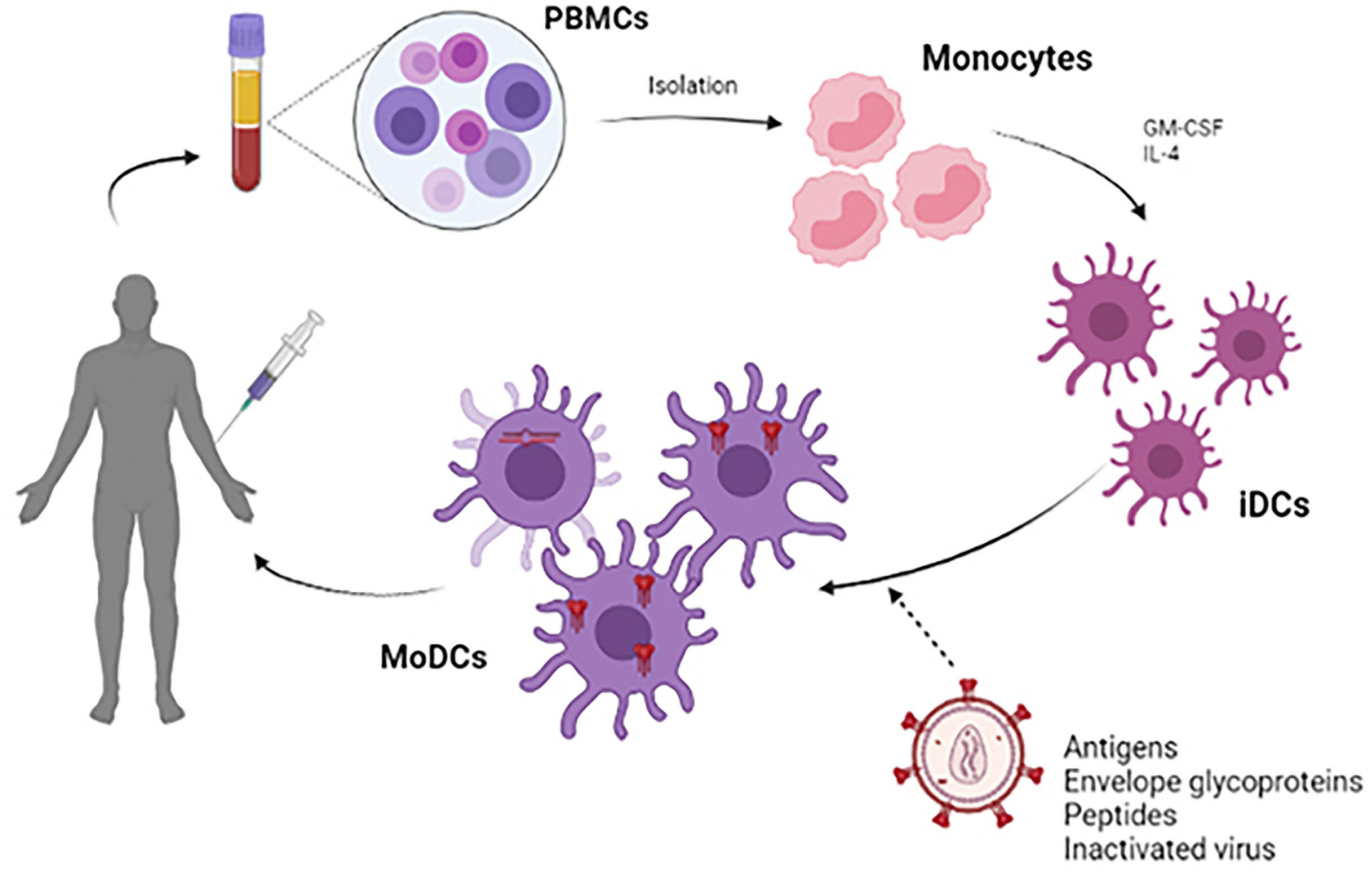
Scheme of the development of a therapeutic vaccine based on DCs. For the development of autologous dendritic cell vaccines, monocytes were extracted from PBMC obtained by leukapheresis from the patient himself. Monocytes are stimulated *in vitro* with GM-CSF and IL-4 to induce differentiation into immature dendritic cells (iDCs). These iDCs are loaded with HIV-1 derived antigen (antigenic peptides, inactivated whole virus, envelope glycoproteins) and will subsequently become mature antigen-presenting dendritic cells. These MoDCs can be used to formulate a vaccine that is administered to the patient to elicit a specific T-cell response to the HIV-1 antigen.

Most DCs vaccines use viral peptides to stimulate autologous DCs and generate a specific cytotoxic T response (77). Accordingly, the protocol to be followed in most cases is based on the extraction and isolation of monocytes from PBMCs treated with GM-CSF and IL-4, deriving these monocytes to immature DCs (iDCs) (78, 79). During the maturation process of these iDCs, antigenic processing occurs, giving rise to monocyte-derived dendritic cells (MoDCs) (80). After that, the adaptive immune response begins with the objective of generating the T cells proliferation. CD4+ T cells will be able to interact with B lymphocytes for the generation of antibodies, while CD8+ T cells will give a cytotoxic response in order to eliminate the infected cells and reduce the viral load, preventing latency. To promote this process, expanding the immune response, MoDCs are capable of secreting a series of inflammatory cytokines such as IL-12p70 and TNFα (15) (**Figure 3** **)**.

DCs vaccines have been developed using different antigens such as DNA vectors, recombinant proteins, and even the attenuated whole virus itself (81, 82).

Some of the first attempts at developing DCs vaccines were based on cancer immunotherapy studies. In these studies, nanotechnology has also been used to load proteins or lysates being used for numerous treatments (NCT02334735) (83) (NCT00045968) (84). Thus, some of these studies show the combination of DCs vaccines with treatments such as chemotherapy (NCT03688178, NCT03657966) (85, 86), radiotherapy (NCT03226236) (87), or agents or immunotherapeutic regimens (NCT03546426, NCT03450044) (88–90). All these studies seem to be a breakthrough and have led to the use of these vaccines for other purposes.

### Immune Checkpoints in DCs Vaccines

There is a number of molecules at the immune level that act as immune checkpoints so that their function is to negatively regulate immune responses to maintain homeostasis. Nowadays, blocking immune checkpoint molecules is considered a strategy to enhance immune responses in patients. Recently, immunotherapy against cancer has attracted much attention due to the successful clinical application of inhibitors targeting the cytotoxic T lymphocyte antigen 4 (CTLA-4) and programmed cell death protein (PD-1)/PD-L1 pathways (91). Studies on cancer vaccines, especially those based on DCs, have made significant progress in recent years. In fact, as mentioned above, the first DCs vaccines were developed as a cancer treatment. In particular, the identification and characterization of the cross-presenting allows effector T cells to mediate immunity against viruses, bacteria, and tumors. In this context, immune checkpoints are often improved during cancer and chronic infections as a mechanism of immune subversion and, therefore, have become a therapeutic target for cancer, but also for the fight against infectious diseases (92).

One of the most important factors that have been studied in recent years is programmed cell death ligand 1 (PDL-1) in DCs and its interaction with PD1 in T lymphocytes (93, 94). This interaction that is established between PDL-1 and PD1 assumes a control of activated T cells. It should be noted that this interaction takes place at two different time points in the life cycle of T cells: First, during the presentation of antigens to naïve T cells for their activation and differentiation, influencing the differentiation pathway of activated T cells leading to cytotoxic, antibody, or regulatory responses. Second, during antigen recognition (95). The PD-1/PD-L1 axis includes the action of effector TL and promotes the depletion of TL, thereby negatively affecting tumor infiltrating lymphocytes in the tumor microenvironment. PD-1 primarily exerts its inhibitory effect on T cells in the periphery where T cells meet PD-1 ligands (96). In cancer, tumor cells and myeloid cells are believed to be the main cell types that mediate T-cell suppression through this junction. In addition, PD-L1 utilizes the STAT3/caspase 7-dependent pathway and directly blocks interferon-γ-mediated cytotoxicity (97). Therefore, blocking the PD-1/PD-L1 axis will have a synergistic therapeutic action when co-administered with DCs vaccine therapy, thus affecting the tumor microenvironment, decreasing IL-10, interferon-γ, and enhancing the function of cytotoxic T lymphocytes (98).

In the case of HIV-1, this interaction between PDL-1 and PD1 has two phases. The first occurs in the early phase of infection where this PD-L1/PD-1 immune checkpoint is low and the CD8 + T cells perform their function by eliminating infected cells. However, in the later, even latent phases of the infection, the expression of PD-L1 is increased by the secretion of different cytokines and acts as a negative feedback system on CD8+ T cells. This circumstance is used by the virus to escape the immune response (99).

The other checkpoint that has been studied is CTLA-4. It was one of the first inhibitory receptors shown to play a role in suppressing T cell responses (100). CTLA-4 is structurally similar to CD28 and binds CD80 and CD86 with a higher affinity than it. In fact, CTLA-4 prevents CD28 binding to CD80/CD86 on DCs, inhibiting the activation of naïve T cells (101). On one hand, CTLA-4 expression by T reg cells serves as a mechanism to suppress excessive T cell responses, while intracellular reservoirs of CTLA-4 prevent tissue damage by T cells spontaneously activated pathogens. In a retrospective study of patients with advanced melanoma whose case progressed after DC vaccine therapy, the addition of ipilimumab (anti-CTLA-4) promoted tumor-specific cytotoxic T-lymphocytic action (102).

Both control points are also fundamental in the case of HIV-1, the use of anti PDL-1, anti PD1, and anti CTLA-4 treatments supposed to improve the immune response that occurs in the T cells and that are fundamental for the good performance of the vaccine based on DCs. However, not all HIV-1 infected patients respond favorably to these inhibitors, possibly due to the lack of immune cells. In addition, therapeutic vaccines have not achieved an adequate CD4+ T response with low antibody levels and poor viral control (103). Perhaps the combination of both, anti-checkpoints and DCs vaccines, can lead to the success of cancer and HIV-1 therapy.

### Homing DCs Vaccine

During activation and differentiation into secondary lymphoid organs, T cells integrate TCR and are exposed to different costimulatory and cytokine signals, inducing the generation of memory and effector cells. The local lymphoid microenvironment is in charge of inducing this differentiation, in addition to selecting the specific properties of tissue localization expression on TLs (104). Localization and chemoattractant receptors can be up-regulated in secondary lymphoid organs and undergo selection during recirculation of cells moving through antigen-rich tissues. The ability to target effector lymphocytes to specific tissues helps increase the efficiency of pathogen clearance and prevents pathological inflammation (105).

Responding T cells program into secondary lymphoid organs and acquire these tissue localization phenotypes within two days of activation, before they leave their initial antigen-encounter site (106). Therefore, the possibility that these phenotypes are induced and randomized during successive rounds of recirculation in peripheral antigen-rich tissues is ruled out. The return of lymphocytes from the blood to the tissues is mediated by a series of sequential interactions between the lymphocyte and the vascular endothelium in specialized postcapillary venules (107).

This entire process is influenced by the pathway of entry of the pathogen into the body, which implies a variation in the availability of molecules that can be processed by dendritic cells at different sites in the lymphoid tissue (108). Some of the most widely studied site-specific phenotypes are those found in the cutaneous versus intestinal secondary lymphoid organs during initial T-cell activation. These would include vitamin A and D derivatives produced by DCs that drain the dermis or intestine positively regulate skin and gut localization markers on T cells (109). DCs can metabolize vitamin D3, a compound that is abundantly present in the skin, transforming it into its active form, and this metabolite suppresses the intestinal localization program in T cells at the same time that it induces the expression of the chemokine receptor, CCR10 (110), which allows localization in the skin. In contrast, dendritic cells located in gut-associated lymphoid tissue can convert vitamin A to retinoic acid. The production of retinoic acid by DCs, particularly those that express CD103, imprints intestinal tropism in T cells by inducing the expression of the integrin α4β7 and the chemokine receptor CCR9 (111–113).

Knowing the homing mechanisms that are established between DCs and TLs are essential when administering a possible therapeutic vaccine. The route of administration of antigen-loaded DCs affects the migration of DCs to lymphoid tissues and the magnitude of the antigen-specific TLs response.

### Approaches in DCs Vaccines

The design of a therapeutic vaccine has two important points in its development. The first is how viral peptides can be introduced into DCs to be recognized as antigens and processed (114). The second is if the administered peptide is capable of stimulating the specific T response after contact with antigen-presenting DCs (115). Some of the most used strategies with the objective of introducing peptides to be recognized as antigens are mentioned below.

One of the ways that has been used to develop a delivery towards DCs has been to conjugate monoclonal antibodies specific for endocytic receptors of DCs, such as DEC205 and Clec9A, with the antigenic particle (116). DEC-205 is a C-type lectin receptor that acts as a recognition receptor for apoptotic and necrotic cells (117). These molecules have been used as a possible target molecule to selectively deliver antigens to DCs because DEC-205 and Clec9A are known to be highly expressed in DCs. Although DEC-205 and Clec9A are promising surface molecules for the targeted delivery of antigens by DCs, vaccines based on these molecules require adjuvants such as anti-CD40 (118) for efficient induction of CD8+ T cell responses. Some of the conjugates that have been used by this technique are those formed by the HIV-1 protein p24 or gag and the monoclonal antibody DEC-205, generating in both cases a B and T response (119). The conjugate with the Gag HIV-1 protein had better results both in the efficacy for the presentation of the antigen, and in achieving a better humoral response, with higher levels of antibodies, as well as cellular (120).

Another strategy that has been used is the formation of a trimer complex formed by the HIV-1 gp140 protein bound to the CD40 ligand (CD40L), incorporating itself directly into the DCs (121). An example of this chimeric strategy is that which was developed from a DNA vaccine encoding the Env protein of HIV-1 together with plasmids encoding the macrophage inflammatory protein alpha1 (MIP-1α) and the tyrosine ligand kinase 3 (Flt3L) (122, 123). This strategy resulted in the recruitment of DCs at the immunization site and induced the expression and maturation of CD11b, CD80, CD83, and MHC class II markers on DCs. One example is found in a DNA vaccine that encodes HIV-1 gp120 bound to the extracellular domain of Flt3L (124). In this case, the expansion of the DCs was induced, but the CD8+ T response was improved and the production of anti-gp120 antibodies increased, remaining up to 16 weeks after amplification of immunization. In addition, CTLA4 or PD-1 have also been incorporated as an alternative for the formation of these complexes as both are important checkpoints as mentioned before (125).

Another approach in recent years is the use of mRNA that codes for TAT, NEF, and REV, incorporating them into the MoDCs of HIV-1 patients. This technique achieves an improvement in the response of TL, both CD4+T and CD8+ T (126). Through the regulation of costimulatory molecules and cytokines, proteins such as Nef and Vpr can affect certain DCs maturation processes or the activation of T cells. The use of specific peptides in this case improves the immunogenic properties in contrast to the use of complete proteins (127).

There are also other types based on the use of the HIV-1 Gag and Env genes incorporating them into an adenovirus that codes for li (invariant chain), ces, rev, vif, and vpr (128). The invariant chain (Ii/CD74) has been identified as a surface receptor for migration inhibitory factor (MIFα). Most cells expressing Ii also synthesize major histocompatibility complex class II (MHC II) molecules, which depend on Ii as a companion and targeting factor. The membrane association of Ii-MHC II complexes allows MIF to target Ii-MHC II to antigen-clustered B-cell receptors (BCRs) and promote BCR-driven signaling and intracellular trafficking. The peptides that will bind to MHC-II are mainly of exogenous origin and are captured by endocytosis to be targeted to lysosomes (129).

In addition, there are also other types of vaccines like DNA vaccines (130, 131) or VLV vaccines (virus like vaccines) (132). Clinical trials have also shown that the current generation of DNA vaccines cannot induce a strong antibody response. Therefore, targeting antigen presentation in the MHC-II pathway to activate CD4 + Т cells seems especially advantageous (133).

However, these approaches are based on the use of the immune mechanisms offered by DCs in prophylactic therapy. DCs are used as intermediaries to generate an immune response, and recent improvements in this area consist in delivering immunogens towards these cells, facilitating their antigen capture and presentation functions through different strategies. Nevertheless, if we focus on the therapeutic use of vaccines based on DCs, in recent years, the use of therapeutic vaccines has been studied with the objective of inducing broad immune responses instead of specific responses of a single antigen to combat viral escape mutants and suppress viral rebound. In this sense, the studies that have been carried out on therapeutic vaccines based on DCs are mentioned below.

AGS-004, was one of the phase IIb trials based on autologous DCs co-electroporated with patient-derived HIV-1 RNA encoding three or four HIV-1 antigens and also CD40L. Although it seemed successful as there was an induction of the CD8+ T response, it was observed that there was no detectable antiviral effect compared to placebo (134). The next trial reached phase I and II using an autologous DCs HIV-1 ApB vaccine. For this, delivery was made with autologous apoptotic cells infected with HIV-1. Although it maintained a good safety profile and produced activation and lysis of infected cells, it did not prevent viral rebound during treatment interruption (135).

A vaccine (DCV-2) composed of autologous myeloid dendritic cells with high doses of heat-inactivated autologous HIV-1 was studied. In those patients in an initial phase of the infection, a reduction in plasma viral load was achieved along with a good T-cell response (136). This suggests that vaccines based on DCs could be used in newly infected patients with a greater chance of success. There is another study completed to phase I in which DCs were used for the delivery of HIV-1 lipopeptides. Despite the polyfunctional response, a virus rebound was observed after 14 days (137).

These studies have not achieved a very successful result, so other alternatives therapeutic vaccines continue to be developed. One of the latest advances is the ALVAC-HIV vaccine that combines the Nef, Pol, Env, and Gag genes of HIV-1 with LIPO-6T (tetanus toxoid class II-restricted universal CD4 epitope combined with 2 Gag, 2 Nef, and 1 Pol peptide) (138, 139). The novelty included in this vaccine is the incorporation of a lipid tail in the C-terminal region that favors the antigenic presentation function (140). In fact, there is a trial of immunized cART-treated HIV^+^ patients with autologous DCs enriched by *ex vivo* culture with GM-CSF and IFN-α and loaded with LIPO5 (2 Nef, 2 Gag, and 1 Pol lipopeptides) (141).

Despite all efforts, effective treatment is still not available with this type of vaccine. This is where nanotechnology plays a very important and advantageous role, both from a prophylactic and therapeutic treatment.

## Nanoparticles in DCs Vaccines

**Figure 4:**
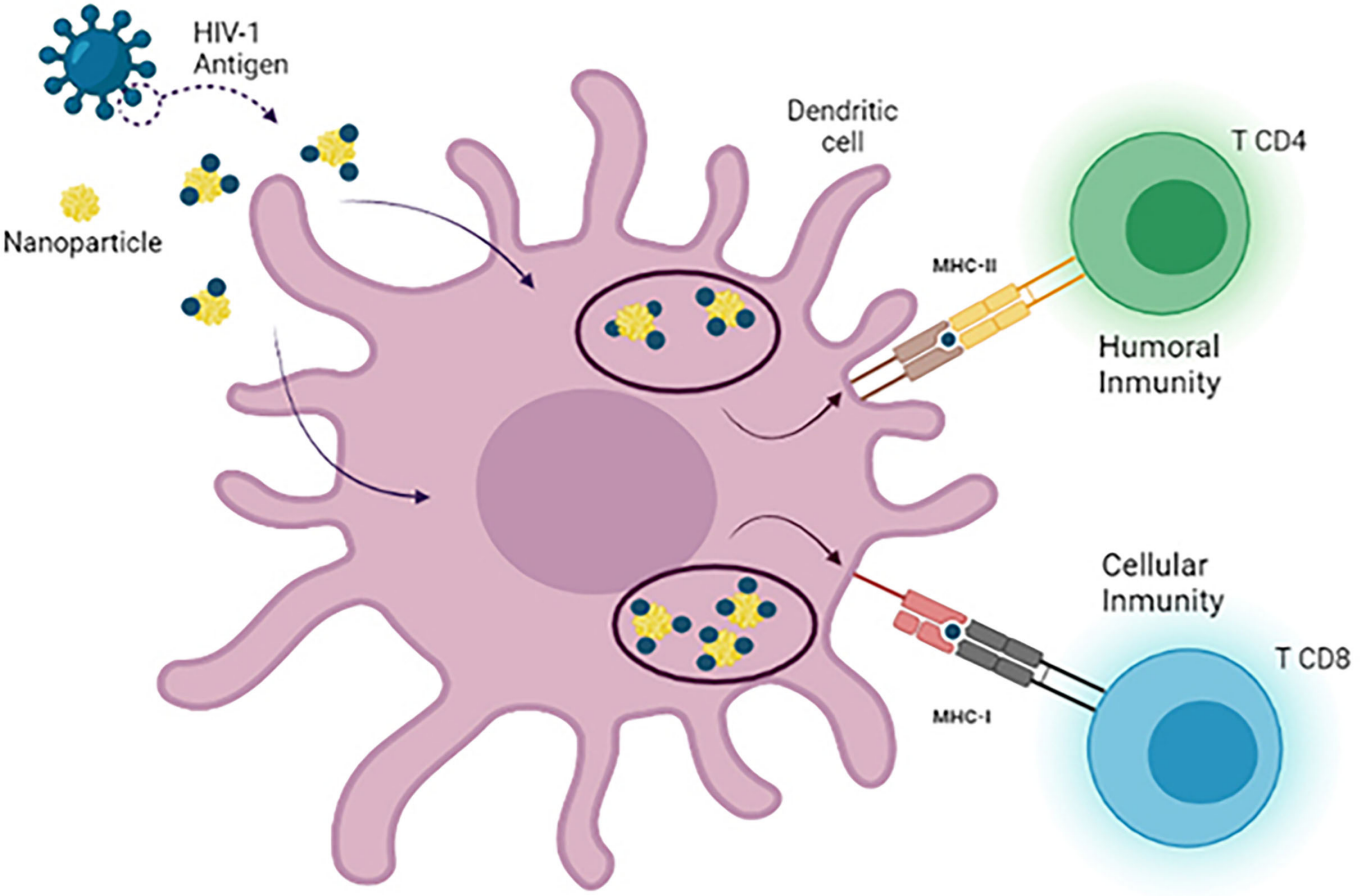
Use of nanotechnology as a delivery system in dendritic cell vaccines. There are different types of nanoparticles that can be used as nanocarriers for antigen delivery in DC-based immunotherapies against HIV-1. During the development of these vaccines, MoDCs are loaded with antigen-nanoparticle complexes that enhance antigen uptake by DCs, leading to the generation of a more specific and potent CD4+ T cell and CD8+ T cell response after reinjection of DCs.

One of the most used approaches in recent years is based on the use of nanoparticles as delivery agents. The size of these nanosystems is an advantage in drug delivery due to their ability to cross physiological barriers, reaching specific cells or intracellular compartments (142). The unique nanodimensional size, symmetrical shape, uniformity, and stable structure of the assembled nanoparticles, which closely resemble native viruses, are all advantages. Notably, smaller nanoparticles (25–40 nm in size) penetrate tissue barriers and traffic to the draining lymph nodes more rapidly than larger nanoparticles (greater than 100 nm in size), which are typically retained by cells at the site of injection and which need to be taken up and trafficked by dendritic cells (DCs) to facilitate their transport to the lymph nodes (143). From this perspective, numerous studies have been carried out in which nanoparticles have been used for the release of small molecules, proteins, or DNA, targeting them specifically to target cells, in this case DCs. Nanoparticles (NPs) are an extraordinary tool to provide different compounds such as DNA, siRNA, and peptides for different cells types (144). Several types of nanoparticles (NPs) such as gold, carbon, dendrimers, polymers, and liposomes nanoparticles have the ability to generate the production of cytokines and antibodies (145). Some of these particles that have been used for these purposes are inorganic NPs (iron and silica) (146), polymeric NPs (chitosan, PLGA, PVPONAlk, γ-PGA) (147), liposomes (cholesterol and lipids) (148, 149), virus-like particles (VLP) (150), and dendrimers (151, 152) **(** **Figure 4** **)**.

DCs need the presence of an adjuvant for their correct activation and maturation. One of the advantages of NPs is that they can encapsulate both the antigen and the adjuvant, thus ensuring the release of both and favoring the establishment of an effective immune response (153). Fortunately, nanotechnology allows new forms of delivery to DCs. For example, studies using liposome-encapsulated HIV-1 gp160 or gp41 proteins or HIV-1 gag p24 protein coated on colloidal biodegradable polylactic acid (poly-d, llactide) could cause activation of MoDCs in patients of HIV-1 and induce specific cytotoxic CD8+ T responses, CD4+ T cell proliferation, and cytokine secretion (154). Polymeric NPs have been highlighted for their use in the development of therapeutic vaccines due to their excellent physico-chemical properties. These particles, in addition to having good safety and biocompatibility profiles, are biodegradable and capable of adjusting on their surface according to requests (155). The most commonly used polymeric NPs for vaccine administration are poly (lactic-co-glycolic acid; PLGA) or poly (lactic acid; PLA) (156). Other polymers of natural origin, such as inulins, alginate, and chitosan, have been used as adjuvants (157, 158).

There is a recent study related to the use of gold nanoparticles *in vivo* that are capable of transporting mannose oligosaccharides that are linked to HIV-1 peptides (159, 160). In this study, the use of these nanostructures used as peptide delivery was demonstrated; in comparison with antigenic peptides alone, they improved the CD4+ T cells and CD8+ T cells response and increased cytokine secretion (161). It seems that the adjuvant mechanism of these mannose ligands is through their binding to type C lectin receptors, such as DC-SIGN, present on DCs (162). As a result, a topical vaccine was developed in the form of a transdermal patch (DermaVir) based on polyethyleneimine mannose (Man-PEI), glucose and a DNA plasmid that encodes the HIV-1 antigen formulated in 100 nms nanoparticles and has reached phase II of clinical trials (163).

Regulating the processing of intracellular antigens is important for antigen presentation and subsequent priming of T cells. DCs have limited lysosomal capacity due to decreased proteolytic degradation, being slower than other APCs (164). The moderate lysosomal capacity of DCs favors the presentation of antigens and gives the opportunity to study another modulation pathway to enhance the degradation and presentation of antigens. Thanks to this mechanism of autophagy, intracellular degradation, and elimination of unneeded or dysfunctional components, the adequate presentation of antigens by DCs occurs, leading to an effective T response. An example is found in the covalent bond between Beclin1, autophagy-inducing peptide, OVA257-264 antigen peptide, and a polymer (165). This nanoconjugate represented an improvement in the induction of autophagy, increasing the efficiency of antigen presentation and the activation of T cells.

One of the nanoparticles that have been used for the design of vaccines are those based on lipids. This can enhance the stimulation of the host’s immune response. Until now, the lipid formulations have been formulated as a unilamellar or multilamellar structure composed by biodegradable phospholipids (phosphatidylserine, phosphatidylcholine, and cholesterol) (166). The mechanism by which these liposomes deliver vaccines is through membrane binding causing internalization of lipid vesicles by DCs (167). The studies that have been carried had lipid particles have been used with those in which phosphatidylserine is conjugated with virus peptides (168).

Other methods, such as the use of dendrimers, have facilitated delivery to dendritic cells. Dendrimers are branched structures that have multiple functional groups at the ends of the terminal branches (169). Thanks to this type of structure, the dendrimer is capable of generating multiple vaccines. A single dendrimer is capable of delivering several components or antigens to the target cell (170). The stimulation capacity of these nanoparticles must be verified to avoid inducing tolerance of the cells (171). Dendrimers, like other nanoparticles, must be capable of inducing the absorption of antigenic peptides by MoDCs and stimulating the immune response.

One of the dendrimeric structures that has been used in order to generate a therapeutic vaccine is the one formed by the glycodendrimers. In a recent study it was shown that these nanostructures can be used for the incorporation of HIV-1 peptides, and although there did not seem to be evidence of cytotoxicity, the glycodendrimers did not modify both the phenotype and some functions of the MoDCs such as the migration capacity and the cytokine profile (172). This is the first study that used a structure formed by the peptide-dendrimer junction forming complexes that are functionalized with maltose as a possible candidate for a DC-based vaccine capable of stimulating the immune system (173). In fact, studies have shown that the presence of maltose and maltosylated macromolecules are capable of binding to langerin, so that cells could potentially be activated by maltose when these nanoparticles are transported by langerin or DC-SIGN due to high structural similarity between them (174).

Another recent study uses G4-70/30 PAMAM polycationic dendrimer and AMC6 nanoparticle derived from β-cyclodextrin as peptide delivery agents (175). In this work, an antigenic peptide derived from the HIV-1 gag p24 protein was used in combination with these polycationic structures, binding to specific receptors. In the case of the G4-70/30 PAMAM dendrimer through mannose (MMR, DC-SIGN), it was linked to clathrin vehicleization, while the cyclodextrin structure could be clathrin-dependent or non-dependent (176). In case of this study, the efficacy of both structures as antigenic carriers was demonstrated, improving the presenting function of DCs and producing the release of pro-inflammatory cytokines IL-2 and TNF-α related to the activation of T cells.

## Summary

HIV-1 infection involves a loss of immune system cells as a consequence of intense immune stimulation that leads to a prolonged and sustained inflammatory state that involves chronic immune activation (177). Controlling this disease state caused by the virus through the use of immunotherapy is a challenge. Therefore, DCs play a major role in the search for a functional vaccine or cure against HIV-1. Their role in the adaptive response, being responsible for the preparation of naïve T cells is a key point, and their manipulation opens the way to new forms of immunotherapy (28). In this sense, the first phase I and II trials in cancer therapy have generated encouraging results (178). Therefore, DCs are also promising candidates in the search for a functional HIV-1 cure or vaccine (69). The objective of the development of a therapeutic vaccine is to induce strong and broad specific immune responses against HIV-1 antigens and that these are capable of resisting viral escape due to mutation in the virus (179). The arsenals available to elicit such responses include DCs as critical mediators of innate and adaptive immunity, as well as proteins, nucleic acids, and viral vector vaccines (180).

Despite all the knowledge about the molecular and physiological mechanisms of both DCs and HIV-1 infection, it is still not known exactly how the response occurs, so more research is needed (181). DCs play a key role due to their ability to serve as a link between innate and adaptive immunity, including in the presence of HIV-1 infection. Knowing in depth the behavior of these cells would allow the design of modulating immune systems that function at different levels, either with a prophylactic or therapeutic purpose (69). To benefit from advantages offered by the field of nanomedicine, different mechanisms have been developed to improve activation by increasing the therapeutic efficacy of DC-based vaccines. Nanotechnology allows DCs activation *in vivo* and simulate natural antigen presentation, favoring lysosomal proteolysis for antigen presentation (182). Additionally, nanotechnology can further intervene in the presentation of antigens, acting directly on the APCs or serving as an adjuvant, improving the efficacy of immunotherapy based on DCs (183). One of the problems with conventional treatments based on DCs is that during the migration of these to the lymph nodes, these therapies can lose activity, being unable to interact with T cells to elicit a better specific response. However, nanoparticles can accumulate in the lymph nodes, which contributes to a high efficiency of lymph node drainage (184). Therefore, nanotechnology offers a promising therapeutic alternative that intervenes in the development of multiple disciplines, signifying great progress in clinical treatment (144).

Despite the fact that an increasing variety of nanoparticles are being directed towards the objective of providing a mechanism to achieve a potent immune response based on the use of DCs, additional studies in the field of modulation of these cells, antigen presentation, and DC-HIV-1 interaction are essential to determinate the foundations for the much desired functional cure of HIV-1 (185).

## Author Contributions

ME-B wrote the initial draft. MM-F edited and corrected the draft, and provided funding. All authors contributed to the article and approved the submitted version.

## Funding

This work has been (partially) supported by the RD16/0025/0019 projects as part of Acción Estratégica en Salud, Plan Nacional de Investigación Científica, and Desarrollo e Innovación Tecnológica (2020-2022) and co-financed by Instituto de Salud Carlos III (Subdirección General de Evaluación), Fondo Europeo de Desarrollo Regional (FEDER), RETIC PT17/0015/0042, and Fondo de Investigación Sanitaria (FIS) 2020-2022 (grant number P119/01638) and EPIICAL Project. This work has been supported partially by a EUROPARTNER: Strengthening and spreading international partnership activities of the Faculty of Biology and Environmental Protection for interdisciplinary research and innovation of the University of Lodz Programme: NAWA International Academic Partnership Programme. This article/publication is based upon work from COST Action CA 17140 “Cancer Nanomedicine from the Bench to the Bedside” supported by COST (European Cooperation in Science and Technology) 2018-2022

## Conflict of Interest

The authors declare that the research was conducted in the absence of any commercial or financial relationships that could be construed as a potential conflict of interest.

## Publisher’s Note

All claims expressed in this article are solely those of the authors and do not necessarily represent those of their affiliated organizations, or those of the publisher, the editors and the reviewers. Any product that may be evaluated in this article, or claim that may be made by its manufacturer, is not guaranteed or endorsed by the publisher.

## Abbreviations

Ce, conserved element; Vif, viral infectivity factor; Vpr, viral protein R; li, MHC class II-associated invariant chain; Rev, regulator of expression of virion proteins; Nef, negative factor; Tat, regulatory protein HIV-1; PLGA, poly (lactic-co-glycolic acid); PVPONAlk, N-vinyl pyrrolidone-ran-propargyl acrylate; γ-PGA, Poly-γ-glutamic acid; Man-PEI, polyethyleneimine mannose; PAMAN, Poly-amidoamine; MMR, manose receptor.
